# SGO: Semantic Group Obfuscation for Location-Based Services in VANETS

**DOI:** 10.3390/s24041145

**Published:** 2024-02-09

**Authors:** Ikram Ullah, Munam Ali Shah

**Affiliations:** 1 Department of Computer Science, City University of Science and Information Technology, Peshawar 25000, Pakistan; ikram.ullah@cusit.edu.pk; 2 Department of Computer Science, COMSATS University Islamabad, Islamabad 45550, Pakistan; 3 Department of Computer Networks and Communication, College of Computer Science and Information Technology King Faisal University, Hufof 31982, Saudi Arabia

**Keywords:** location privacy, location-based services, pseudonyms, VANETs, anonymizations, location obfuscation

## Abstract

Location privacy is an important parameter to be addressed in the case of vehicular ad hoc networks. Each vehicle frequently communicates with location-based services to find the nearest location of interest. The location messages communicated with the location server may contain sensitive information like vehicle identity, location, direction, and other headings. A Location-Based Services (LBS) server is not a trusted entity; it can interact with an adversary, compromising the location information of vehicles on the road and providing a way for an adversary to extract the future location tracks of a target vehicle. The existing works consider two or three neighboring vehicles as a virtual shadow to conceal location information. However, they did not fully utilize the semantic location information and pseudonym-changing process, which reduces the privacy protection level. Moreover, a lot of dummy location messages are generated that increase overheads in the network. To address these issues, we propose a Semantic Group Obfuscation (SGO) technique that utilizes both location semantics as well as an efficient pseudonym-changing scheme. SGO creates groups of similar status vehicles on the road and selects random position coordinates for communication with the LBS server. It hides the actual location of a target vehicle in a vicinity. The simulation results verify that the proposed scheme SGO improves the anonymization and entropy of vehicles, and it reduces the location traceability and overheads in the network in terms of computation cost and communication cost. The cost of overhead is reduced by 55% to 65% compared with existing schemes. We also formally model and specify SGO using High-Level Petri Nets (HLPNs), which show the correctness and appropriateness of the scheme.

## 1. Introduction

Vehicular Ad Hoc Networks (VANETs) have an important role in enhancing road safety and traffic efficiency by using communication links between different entities of the road network [1]. The basic safety message or beacon is broadcast in the network to disseminate road status information. There are two basic communication models, i.e., vehicle-to-vehicle (V2V) and vehicle-to-infrastructure (V2I) models to provide various services and facilitation to vehicle drivers [2]. The services may be location services, games, advertisements, news, email services, etc. V2V facilitates communication between vehicles, while V2I provides communication between vehicles and infrastructure for exchanging road context information in the network. The basic technological types of equipment are On-Board Units (OBU), sensors, Event Data Recorders (EDRs), radars, cameras, omnidirectional antenna, Global Positioning System (GPS), etc. [3]. Dedicated short-range communication (DSRC), cellular networks, WiMax, WiFi, and VeMac are the wireless communication technologies utilized in VANETs [4].

There are two main categories of VANETs applications: safety-related applications and comfort or infotainment applications. Safety application includes road accident information, emergency, lane-changing warning, collision avoidance, and so on. The comfort applications contain location service facilities, weather information, advertisements, games, and so forth [3]. Location-Based Services (LBSs) are increasing in popularity with the emergence of GPS in mobile devices. For the nearest location of interest, a vehicle is required to communicate with the location server. Although an LBS server provides benefits to society and users, it creates serious privacy issues for service consumers [5]. Each vehicle must share its current/physical location with the location server to obtain location services facilities [6]. If the location of a vehicle is obtained by a malicious entity that causes serious threats to the driver, the threats may be damage to social reputation, property loss, victim of blackmail, and physical harassment [7].

To handle location privacy problems, several techniques are proposed: mix zone schemes [8,9,10], silent period based techniques [11,12,13], and path confusion approaches [14,15,16]. A mix zone is an area where several vehicles gather and mix their identities. That hides the actual identity of vehicles in the concerned region. However, it is difficult to provide privacy under lower traffic conditions in the Mix zone. In silent period schemes, vehicles remain silent for a certain amount of time in order to not broadcast safety messages. These schemes impact safety-related applications. Path-confusion techniques take the help of dummy location data to hide the location information of vehicles. The path confusion creates extra overhead in the network while generating dummy data. The mentioned existing techniques are mostly adapted for broadcasting and sharing location with neighboring vehicles, and communication with the location server is not fully utilized.

One of the location privacy schemes presented in Ref. [17] for preserving the location information of vehicles communicating with the LBS server has certain limitations and problems. Firstly, each vehicle selects four random position coordinates in connection with its neighbors, which increases computation cost. Secondly, a vehicle sends four location messages to the LBS server, which increases communication delay. Thirdly, for higher traffic density, a lot of dummy location messages are generated that impact road network applications. Fourthly, in the case of low density—for example, a single vehicle is moving on the road—there will be four messages transmitted to the LBS server so the adversary may analyze the four location coordinates with the same speed and timestamp in each message and can easily identify the target vehicle in the vicinity. Similarly, a location privacy scheme [18] takes a neighboring vehicle as a virtual shadow to send multiple requests to the LBS server for hiding the actual location of a target vehicle. However, these requests contain the same pseudo-identity and timestamp that provide a way for an adversary to identify a target vehicle.

To address the mentioned location privacy problems, in this paper, we propose a Semantic Group Obfuscation (SGO) scheme that provides location protection and identity hiding while communicating with the LBS server. The proposed scheme takes neighboring vehicles’ semantic information to make a group of vehicles based on transmission range in a vicinity. Random position coordinates are taken on the road to be put in the location messages. These messages are forwarded to the location server, which hides the location of each vehicle in the group. We also use a pseudonym-changing mechanism that changes the pseudonyms of vehicles before exchanging location messages with the LBS server. Certainly, this scheme provides both location and identity protection. Here, we used various terms in the paper such as target vehicle, beacon, and location message. The location of the target vehicle is important for an adversary to identify. A beacon is a message which is broadcast in the network for gaining road environment information. The location message is used for communication with the LBS server to access the nearest location of interest. Our contributions to this research work are outlined below:

- We introduce the concept of semantic location obfuscation mechanism for information hiding. One of the locations is selected randomly in different distance ranges of the road network. This location is included in the location messages of each group member that hides actual location information.
- The proposed scheme takes a single location message for communication with the LBS server that reduces the cost of computation and communication compared with existing schemes.
- We conduct a formal modeling of the SGO using HLPN. It verifies the validity of the proposed scheme and shows the correctness of data flow during the processing of the scheme.

The rest of the paper is organized as follows. The literature review is discussed in Section 2. Section 3 has details about the system model and the adversary model. The proposed scheme is explained in Section 4. We also formally analyzed the scheme in Section 5 using high-level Petri nets. Section 6 has an experimental evaluation that provides comparative results with existing schemes. The analysis and discussion in Section 7 contain privacy protection against adversaries, impact on location service utility, algorithm complexity, and cost of computation and communication. Finally, the paper is concluded in Section 8.

## 2. Related Work

A vehicle exchanges location messages with the LBS server to obtain their nearest location of interest. The location message contains the actual/physical location of a vehicle. This location may be used by an adversary to extract the future location tracks of vehicles that may harm a vehicle driver. To solve the mentioned location privacy issue, several techniques are proposed in the literature. We highlighted some of the important techniques regarding privacy problems in this section.

Endpoint zones are created in Ref. [19], where vehicles remain silent to hide user location. Vehicles in the zones can share login credentials. The transmission silence creates confusion for an adversary to know the activity of vehicles in the group. In Ref. [20], multiple zones are suggested to be deployed for privacy protection. The multiple zones are deployed by using road network traffic conditions, which improved privacy protection levels. Another multiple mix zones concept is used in Refs. [21,22] for the preservation of location privacy. In this technique, a user defines a path by selecting two endpoints on the map service by not disclosing secret information. The path communicated to map services is slightly changed to ensure privacy and quality of service. Similarly, a de-correlation multiple mix zone region is deployed for parking lots and traffic jam places [8], where the privacy of vehicles is important while moving toward the destination. For vehicles that stop at traffic jams or parking lots, a mix zone is automatically created, and all vehicles change pseudonyms to a de-correlate link between old and new pseudonyms. To mix the context of vehicles moving on the road, a context-based mechanism is introduced in Ref. [9] that provides privacy protection against a global passive adversary. In Ref. [10], vehicles having the same mix context cooperatively swap and change pseudonyms without infrastructure support. For accountability, the exchange of pseudonyms is reported to the authority. A common pseudonym-changing approach is presented in Ref. [23] for the location protection of vehicles, in which all neighboring vehicles used one common pseudonym certificate for a short period before changing their individual pseudonyms. However, this paper does not address the misbehaving vehicles in the region. The location privacy schemes taking the concept of a mix zone have certain issues and problems: firstly, it is difficult to provide location privacy in lower traffic conditions; secondly, privacy is protected in the zone only, and outside of it, no protection is offered.

The concept of a random silent period is used in Ref. [24] where vehicles update pseudonyms during silence, which breaks the link between the old and new pseudonyms. The grouping of vehicles prevents location tracking from the location service provider. In Ref. [25], vehicles become silent when speed drops to a certain threshold. Each vehicle changed pseudonyms during the silent period. The synchronized pseudonym changing hides the actual identity of vehicles. A context-aware location privacy scheme based on a silent period is introduced in Ref. [26], which allows vehicles entering a silent period to change their pseudonym adaptively using context information such as finding silent neighboring vehicles. Another location unlinkability mechanism is presented in Ref. [27] that consists of two methods, i.e., the pseudonym-changing process and cheating detection mechanism. The cheating detection mechanism first detects malicious vehicles in the region which launch cheating attacks. The group of vehicles is permitted, and the pseudonym is changed in silent mode. Similarly, an alternative technique of cooperative pseudonym exchange and scheme permutation is proposed in Ref. [11] for location privacy in VANETs. Vehicles cooperatively exchange their pseudonyms to prevent location tracking from service providers. Likewise, in Ref. [12], a new cooperative pseudonym-changing scheme is offered in which vehicles change their identities in a synchronous way during a silent period. Overseeing vehicles are arranged in Ref. [13] that monitor the road environment and let other vehicles join a silent period, which ensures the safety as well as privacy of vehicles. A safety-aware scheme is introduced in Ref. [28] where vehicles have two statuses, i.e., silent and active. In the silent status, each vehicle remains silent, while in an active state, vehicles broadcast safety-related messages. During the silent period, each vehicle monitors neighboring vehicles for accident situations; if this occurs, the vehicle exits the silent period and shares the neighbor’s location. Conversely, the monitoring of neighboring vehicle locations provides a way for an adversary to track the concerned vehicle. The main drawback of silent period schemes is that they impact safety-related applications: for example, if a road incident occurs and vehicles are silent during this time, how this event could be disseminated in the network?

A path confusion scheme is introduced in Ref. [29] for the location privacy of vehicles. The protection is provided by exchanging the reported position of two users, which increases the confusion of an adversary about the actual location of a communicating entity. Privacy by the decoying method is introduced in Ref. [30] that takes the help of dummy or false queries in connection with other vehicles on the road to cover up an actual location from a global passive adversary. Similarly, the privacy of a vehicle is preserved [31] by the generation of the virtual location of surrounding vehicles dynamically for confusing driving routes. In Ref. [32], a circle-based dummy generation algorithm is proposed for dual location privacy. The scheme provides privacy to vehicles at low computation and communication costs. Likewise, dummy location selection algorithms are given in Refs. [14,15], respectively. A mutually obfuscating paths method is introduced in Ref. [16] in which vehicles generate plausible location updates for each other to divert the viewpoint of the LBS server from a target vehicle’s actual location. The vehicle’s location is randomized with the help of differential privacy using reinforcement learning [33], which protects the location trajectory. The obfuscation policy is optimized using reinforcement learning, which reduces privacy risk and does not affect service utility.

Trust management is considered an important factor for the evaluation of the trustworthiness of vehicles in VANETs. Each vehicle should verify the reliability of received messages from other vehicles in the network. Trust management can be used at the same time as privacy preservation schemes in vehicular networks to achieve efficient results regarding privacy protection. In Ref. [34], a trust management solution is provided which creates a balance between trust and privacy preservation in the VANETs. A fully distributed context-aware trust model is introduced in [35] to improve the reputation model for service recommendation. The recommendation operation is directly performed by the service provider. The service consumer considers various factors such as number, context weight, time decay, and preference to calculate the trust of the service provider. Similarly, a reputation-based privacy-preserving model is present in Ref. [36]. It takes the help of elliptic curve cryptography and paillier algorithms in which the calculation and processing of reputation feedback are completed by a cloud service provider. The scheme proposed in Ref. [37] takes various behavior attributes of participants for trust calculation. The system accurately computes the trustworthiness of participants with diverse behavior patterns. This identifies various types of behavior attacks that provide a way for service consumers to hide personal information from an adversary.

A virtual trajectory approach is introduced in Ref. [38] that utilizes virtual points according to the user’s need to make virtual trajectories. This creates a bridge between a user and the LBS server which provides efficient privacy protection. The privacy of user queries is preserved using the oblivious transfer extension protocol in Ref. [39], which is efficient regarding computation and communication costs. An anonymous area is created in Ref. [40], taking anonymous neighboring vehicles to obtain dummy locations of requested vehicles in a different context, which strengthened the location privacy of vehicles. To mislead an attacker, a target vehicle selects a shadow of other vehicles to generate multiple virtual trajectories for communication with the LBS server [18]. Likewise, in Ref. [41], shadow vehicles are selected using the deviation of predicted trajectory, and then fake queries are put into the actual and shadow vehicles’ queries. Another location service query-based location privacy scheme based on the ring signcryption method is proposed in Ref. [42], where vehicles anonymously connect with a location server that provides both query and data privacy. A cloaking region obfuscation scheme is developed in Ref. [43], where vehicle identity and location are indistinguishable in the region of interest. A vehicle location trajectory is protected using caching and dummy positions [44] while communicating with the LBS server. in Ref. [45], a collaborative trajectory obfuscation privacy scheme is proposed in which a vehicle takes the help of the Kalman filter algorithm to select collaborators for future location prediction. Those collaborators are selected who can mislead the adversary about a vehicle’s actual position.

After a detailed discussion of the existing location privacy schemes, it is important to acknowledge that there are certain problems in the existing research work. The mix zone location privacy scheme makes it difficult to provide privacy under lower vehicle traffic conditions, and it only offers privacy within the zone; outside it, there is no protection. The silent period techniques offer location protection; however, they impact safety-related applications. Dummy or location confusion techniques provide location privacy at the cost of higher computation and communication costs. Location protection schemes [17,18] have taken multiple neighboring vehicles as shadows for location protection; however, they create extra overhead for dummy data generation in the network. Moreover, they did not use a suitable pseudonym-changing process that provides a way for an adversary to link the pseudonyms of vehicles at different locations. Therefore, we propose a new semantic location obfuscation mechanism that addresses the mentioned issues and problems. The proposed mechanism selects random position coordinates and an efficient pseudonym-changing process which provides both location and identity protection. Furthermore, our scheme exchanges a lower number of location messages with the LBS server, which reduces communication and computation costs.

## 3. Models and Goals

This section contains details of models and goals the research work. The models consist of the system model and the adversary model.

### 3.1. System Model

**Figure 1:**
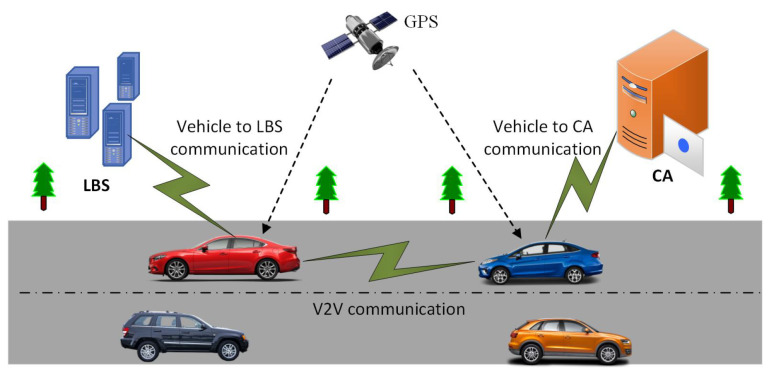
System model.

The system model consists of three entities including vehicle, Certification Authority (CA), and Location-Based Services (LBS), which are shown in Figure 1. Before the usage of the network, each vehicle must be registered with CA. CA is a trusted entity that cannot take part in compromising the location information of vehicles. It provides certificates to vehicles at the time of registration. The certificate consists of public key $PK_{i}$ and a set of pseudonyms *P*. All vehicles on the road have GPS receivers, and real-time location information is received through GPS. The LBS server is not a trusted entity and can take part in compromising vehicle location information. A vehicle requires a location query to the LBS server for the nearest location of interest. Here, we take two types of messages, i.e., beacon and location message. The beacon format is $V_{ID},POS,V,D,T_{x},NeighCount,otherheadings$ where $V_{ID}$ is vehicle identity, POS is vehicle location, *V* is the speed of a vehicle, *D* is the moving direction, $T_{x}$ is the transmission range and NeighCount is neighbor counting in the transmission range. The beacon is broadcast to collect road network information and used in the neighbor function to count the number of neighboring vehicles in the transmission range. We consider the location message format as PseudoID,SemPOS,D,T,Sig. In the location message, PseudoID is a pseudonym, SemPOS is a semantic location taken from the road network, *D* is the direction, *T* is a timestamp, and Sig is a signature used for authentication services.

### 3.2. Adversary Model

**Figure 2:**
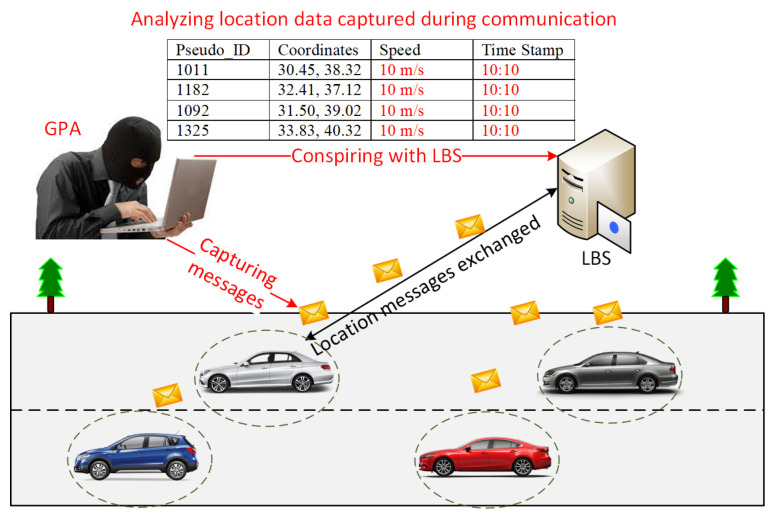
Adversary model scenario.

In this research, we consider a Global Passive Adversary (GPA), which may try to identify a target vehicle in the road network. The GPA can cover a large part of the road network. The adversary can capture location messages exchanged with the LBS server and try to analyze these messages for the identification of a target vehicle in the vicinity, as shown in Figure 2. According to the figure, the location message contains vehicle identity, position coordinates, speed, timestamp, and other headings. The GPA captures these location messages during communication with the LBS server. The location messages are analyzed by GPA to extract the location and identity information of a target vehicle. The adversary tries to match and link the captured information with old data and comes to know the behavior of vehicle drivers that may be connected with a bank or have important political persons. This creates several dangers to the vehicle driver such as blackmailing, property loss, social defamation, etc., [3,46]. In this research, we consider the following assumption about GPA strength.

1. GPA can capture vehicle location messages during communication with the LBS server.
2. GPA can analyze the location messages for vehicle identity and locations.
3. GPA can apply pseudonyms linking attack

## 4. Proposed Solution Semantic Group Obfuscation (SGO)

**Figure 3:**
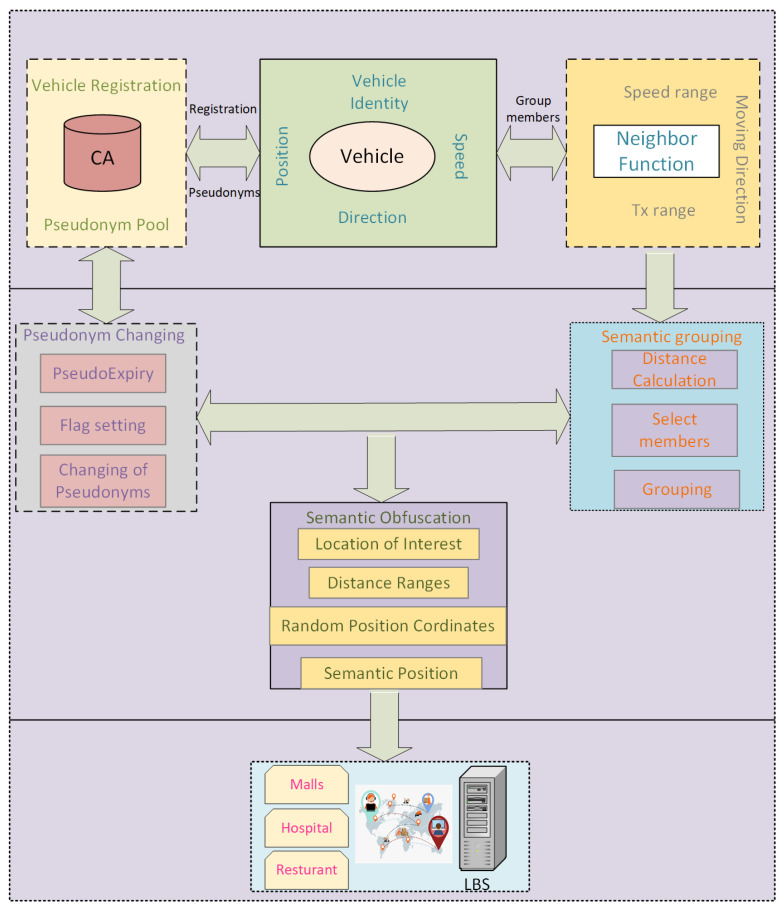
Block diagram of the proposed scheme.

In this section, we introduced our proposed new location privacy scheme called “Semantic Group Obfuscation (SGO)” for vehicular networks. A vehicle takes the help of the nearest neighboring vehicles in the transmission range. A group of transmission range vehicles is formed based on the same speed range, location, and direction. Vehicles take coordinates of the well-known location in a group manner. Each vehicle in a group includes this location in the message communicated with the LBS server instead of the actual location. The block diagram of the proposed scheme is shown in Figure 3. Each vehicle must register with CA before joining the network, and a pseudonym pool is assigned to vehicles. Vehicles use the neighbor function to collect information about neighboring vehicles, which is given in Algorithm 1. This algorithm counts the number of neighboring vehicles in a transmission range of 500 m. A group of vehicles is formed using location semantics in a road network. After that, the pseudonyms-changing process is applied. Before communicating with the LBS server, the semantic obfuscation method is used for hiding the location information of vehicles. The components of the proposed scheme are explained in the following subsections. The nomenclature used in the paper is given in Table 1.

**Algorithm 1** Neighbor Function**Initialization**: $V_{i}$: Any vehicle i, $SP_{R}$: Speed range, D: Direction, $V_{ID}$: Vehicle identity, CountID: Counting number of vehicles, MessageBroadcast: Broadcast of messages, DistanceCalculate: Calculation of distance between neighboring vehicles, DistanceRange: Transmission range, CheckLimit(!Expiry): Checking of neighbors search limit. **Input**: $SP_{R},D,V_{ID}$ **Output**: Counting of vehicles (CountID)
   1: **for** all $V_{i}=1\to n$ **do**   2:    MessageBroadcast(i)   3:    Check($SP_{R},D,V_{ID}$)   4:    $DistanceCalculate(V_{i},V_{j})$   5:    **if** $V_{ID}\neq V_{ID}\left(i\right)$ && DistanceRange≤ 500 m **then**   6:    CountID++   7:    **else**   8:    CheckLimit(!Expiry)   9:    **end if** 10:   **end if** 11: **end for** 12: **end for** 13: Return (CountID)


### 4.1. Working of SGO Scheme

In the semantic obfuscation method, a vehicle will use neighboring vehicles to obfuscate its location. For this purpose, an initiator vehicle will search neighbors in its transmission range and form a semantic group. Afterward, the initializing vehicle takes three different distance ranges $R_{1}$, $R_{2}$, and $R_{3}$. The distance ranges consider different distances in meters: $R_{1}$ is in 1–100 m, $R_{2}$ is in 101–200 m and $R_{3}$ is in 201–300 m. In each range, the location coordinates of a place are taken randomly. Three different position coordinates are taken in these ranges. After computing all the distance ranges and position coordinates in the vicinity, one of the position coordinates is taken randomly from these three ranges: $R_{1}$, $R_{2}$, and $R_{3}$. The selected position coordinates are adapted by each group member in the location message, and a location query is sent to the LBS server with the same position coordinates. This location query hides the actual location information of each vehicle in that region. Likewise, different semantic groups may be made in various regions of the road network. The semantic obfuscation procedure is given in Algorithm 2. The “For” loop (line 1) takes every vehicle in a vicinity that requires the location of interest (line 2), lines 3 and 4 compute the neighbor function and semantic grouping, distance ranges are calculated in line 5, and lines 6–16 select position coordinates randomly in different distance ranges; one of the positions is selected in line 15, and semantic location is included in the location message and sent to the LBS server (lines 19–22).

**Algorithm 2** Semantic obfuscation**Initialization**: $V_{i}$: Any vehicle i,R: Distance range in meters, LoI: Location of interest, RandPOS: Random position, SemPOS: Semantic position, PR: Position coordinates in ranges, LocMessage: Location message. **Input**: Distance ranges $R_{1},R_{2},R_{3}$ **Output**: selection of semantic location   1: **for** all $V_{i}=1\to n$ **do**   2:    RequireLoI   3:    NeighborFunction()   4:    Semanticgroup()   5:    Calculate Distance ranges   6:    **if** DistRange≤ 100 m **then**   7:    Search Position coordinates   8:    Select $PR_{1}$ Randomly   9:     **end if** 10:    **end if** 11:    **if** DistRange in 101–200 m **then** 12:    Search Position coordinates 13:    Select $PR_{2}$ Randomly 14:    **end if** 15:    **end if** 16:    **if** DistRange in 201–300 m **then** 17:      Search Position coordinates 18:    Select $PR_{3}$ Randomly 19:   **end if** 20:   **end if** 21: **end for** 22: **end for** 23: $SemPOS\left(P\right)=RandPOS\left(PR_{1},PR_{2},PR_{3}\right)$ 24: Pseudonym−changing() 25: $LocMessage[V_{i}\left(PseudoID,SemPOS\left(P\right)\right),otherheadings]$ 26: Send query (LocMessage()) to LBS 

### 4.2. Semantic Grouping

**Figure 4:**
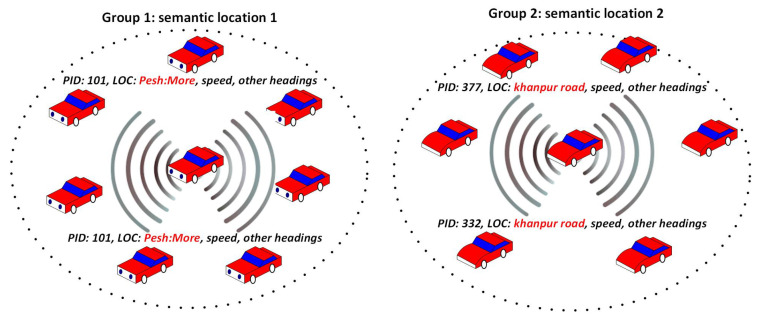
Semantic group obfuscation concept.

A group of vehicles with similar speed range, direction, and transmission range is considered a semantic group. One of the vehicles is selected randomly by CA as a group initiator among transmission range neighboring vehicles just like given in Ref. [3]. The initiator vehicle has the responsibility of the management of members in a group, semantic location, and pseudonyms changing. TA verifies the identity of the initiator vehicle, and other vehicles in the transmission range request for the initiator to join the group. The grouping of vehicles starts with the counting of neighbors with a minimum distance range. Vehicles within small distance ranges are combined to make a group. Here, NeighThreshold is used to control the number of members in a group (the neighbor threshold contains the number of vehicles between 24 and 31, which is considered high traffic density according to [47]). If the number of vehicles is increasing in a group beyond the threshold, then the ReducedNeigh(limit) disjoins some vehicles from the group to reduce the burden in a group. The detailed procedure is shown in Algorithm 3. The “For” loop (line 1) takes all the vehicles in the region; in line 2, CA selects the group initiator vehicle; line 3 takes the help of the neighbor function algorithm to compute the number of $T_{x}$ neighboring vehicles; the distance among the neighbors is calculated in line-4; and the neighboring vehicles with minimum distance ranges are included in the semantic group (lines 5–9), lines 10–11 restrict the number of vehicles in a group according to a neighbor threshold. A group of vehicles is formed, and Algorithm 3 returns the number of members in that group. The concept of semantic grouping and location obfuscation is shown in Figure 4. Two groups of vehicles are made on the road within the transmission range. The members of Group 1 contain the semantic location “Peshawar more” in the location field, while Group 2 members show “Khan pur road”. This will hide the actual positions of each member’s vehicles in a group.

**Algorithm 3** Semantic Grouping**Initialization**: $V_{i}$: Any vehicle *i*, $T_{x}$: Transmission range, SemGroup: Semantic grouping, CalculateDist: Calculation of distance ranges with neighbors, Distance(min): Check neighbors with minimum distance, NeighCount: Count neighbors with minimum distance, NeighThreshold: Neighbor threshold, SemGroup(i): Making the group of vehicles *i* with semantic location, AddNeighbors(i): Adding min distance neighbors *i* in a group, ReducedNeigh(limit): Reduction of members from a group with some limit. **Input**: Number of vehicles in $T_{x}$ **Output**: A group of vehicles   1: **for** each vehicle $\left(V_{i}\right)$ **do**   2:   Initiator selection by CA   3:   $NeigFunction(SP_{R},T_{x},D)$   4:   $CalculateDist(V_{i})$   5:   Distance(min)   6:    **for** Vi∈Distance(min) **do**   7:    NeighCount(min)   8:    **if** NeighCount≤NeighThreshold **then**   9:      SemGroup(i)=AddNeighbors(i) 10:   **else** 11:      SemGroup(i)=SemGroup(i)−ReducedNeigh(limit) 12:     **end if** 13:     **end if** 14:   **end for** 15:   **end for** 16: **end for** 17: **end for** 18: Return(SemGroup(i))


### 4.3. Pseudonym Changing Process

The pseudonym-changing process is important to hide the actual identity of a vehicle. Before sending a location message to the LBS server, a vehicle should change its existing pseudo-identity to a new one. For this purpose, a vehicle will set a flag to 1, which means it is ready to change its pseudonym. Each member of the group will receive the position coordinate of a random place to communicate with the LBS server. Then, all the group members will change their pseudonyms and include new pseudonyms and location information in the location message. After changing the pseudonym, the flag is set to 0, which means the pseudonyms of vehicles in the group were successfully changed. The detail is given in Algorithm 4. The for loop (line 1) is used for each member of the semantic group (line 2), pseudonym expiry is checked in line 3, in line 4, the flag is set to 1, message broadcast is completed in lines 5–9, the pseudonyms of all vehicles are changed in line 10, and the newly changed pseudonyms are returned in line 12.

**Algorithm 4** Pseudonym Changing**Initialization**: $V_{i}$: Any vehicle *i*, PseudoExpiry: Pseudonym Expiry, MsgBroad: Message Broadcast, PseudoIDs: Pseudonym identities of vehicles, $Change(PseudoIDs\left(V_{i}\right))$: Changing pseudonyms of vehicles. **Input**: PseudoExpiry,SemPOS **Output**: Assign new pseudonyms to vehicles   1: **for** vehicle $V_{i}\in Group\left(j\right)$ **do**   2:    SemanticGrouping()   3:    Check PseudoExpiry   4:    Set flag to 1   5:    **if** $V_{i}$gets(SemPOS) **then**   6:    MsgBroad(PsuedonymChange)   7:    **else**   8:    Go to step 2   9:    **end if** 10:   **end if** 11:   Change(PseudoIDs(Vi)) 12:   Set flag to 0 13:   Return(Vi(PseudoIDs)) 14: **end for** 15: **end for**


## 5. Formal Modeling

**Figure 5:**
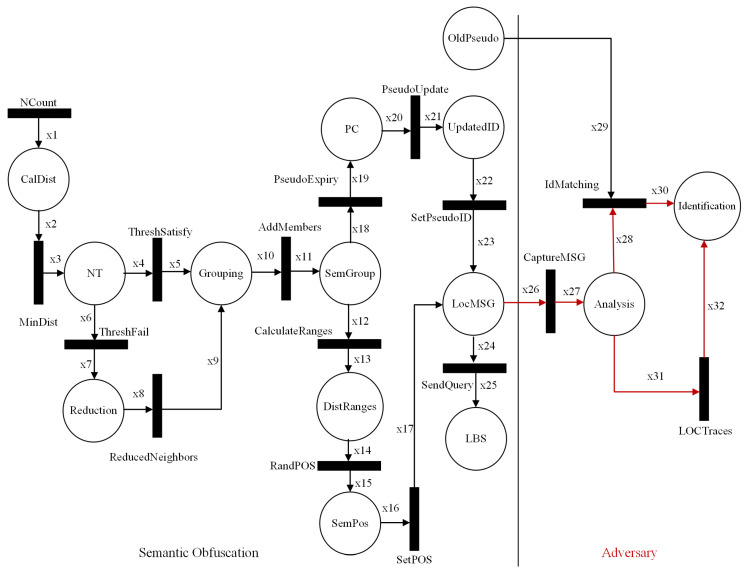
Adversary analysis on semantic obfuscation scheme.

High-level Petri nets (HLPNs) are used for two reasons [4]: to simulate the proposed scheme and present mathematical modeling to analyze the structure properties and behavior of the scheme. The benefits are that it can verify the interconnection of system components and processes, information flow, and processing. We formally model our proposed scheme in HLPN, and it consists of seven tuples: $\left(P,T,F,\varphi ,R,L,M_{0}\right)$, as defined in Ref. [48]. The HLPN of the SGO privacy scheme is shown in Figure 5. In HLPN, we present the proposed scheme in terms of mathematical rules/properties. Table 2 contains details of the symbols used in HLPN. We define places that specify the set of rules in formal modeling, as shown in Table 3.

As shown in the figure, we started the HLPN from the neighbor function. The neighbor function is used to count the number of neighboring vehicles in a vicinity. Vehicles to be included in a group required neighbors with minimum distance ranges. The neighboring vehicles with minimum distance are calculated in Equation (1). After it, the neighbor threshold is verified. Equation (2) shows the satisfaction of the threshold and is updated accordingly; otherwise, the procedure will move toward Equation (3) in case of failure. If the number of vehicles is higher than NeighThreshold, then some of the vehicles are discarded from joining the group, as given in Equation (4).

(1) $$\begin{array}{c} R(\mathrm{MinDist})=\forall \;i2\in x2\wedge i3\in x3\;|\;i2[2]<i3[2]=True\\ \wedge x3^{\prime }:=x3\cup \left\{i3\left[2\right],i3\left[3\right]\right\}. \end{array}$$

(2) $$\begin{array}{c} R(\mathrm{ThreshSatisfy})=\forall \;i4\in x4\wedge i5\in x5\;|\;(i4[3]<=i5[3])=\\ satisfy\to x5^{\prime }:=x5\cup \left\{i5\left[4\right]\right\}. \end{array}$$

(3) $$\begin{array}{c} R(\mathrm{ThreshFail})=\forall \;i6\in x6\wedge i7\in x7\;|\;(i7[2]\in i6[1]\wedge i6[3]>i7[3])=True\\ \to x7^{\prime }:=x7\cup \left\{i7\left[4\right]\right\}. \end{array}$$

(4) $$\begin{array}{c} R(\mathrm{ReducedNeighbors})=\forall \;i8\in x8\wedge i9\in x9\;|\;(i9[2]\in i8[1]\wedge \\ x9^{\prime }:=x9\cup \left\{i9\left[4\right],i9\left[5\right]\right\}. \end{array}$$

The vehicles are added to the semantic group based on the threshold, as shown in Equation (5). Now, it is time for semantic location collection. Three distance ranges in meters are calculated in Equation (6). One of the position coordinates is selected randomly, as shown in Equation (7). The selected location is included in the location message, as given in Equation (8).

(5) $$\begin{array}{c} R(\mathrm{AddMembers})=\forall \;i10\in x10\wedge i11\in x11\;|\;i11[2]\in i10[1]\wedge Add(i11[3])\\ \to (x11^{\prime }:=x11\cup \left\{i11\left[4\right]\right\}). \end{array}$$

(6) $$\begin{array}{c} R\left(\mathrm{CalculateRanges}\right)=\forall \;i12\in x12\wedge i13\in x13\;|\;\left(i13\left[2\right]\in i12\left[4\right]\right)\to x13^{\prime }:=\\ x13\cup \{i13[3],i13[4],i13[5]\}. \end{array}$$

(7) $$\begin{array}{c} R(\mathrm{RandPOS})=\forall \;i14\in x14\wedge i15\in x15\;|\;(i15[2]\in i14[4])\wedge \\ Rand(x15^{\prime }:=x15\cup \left\{i15\left[3\right]\right\}. \end{array}$$

(8) $$\begin{array}{c} R(\mathrm{SetPOS})=\forall i16\in x16\wedge i17\in x17|\;(i17[1]\in i16[3])\wedge \\ Set(x17^{\prime }:=x17\cup \left\{i17\left[4\right]\right\}). \end{array}$$

The validity of the pseudonym of vehicles is checked in Equation (9). After it, the pseudonym update process is started where the flag is set to 0, meaning the vehicles change pseudonyms successfully, as shown in Equation (10). The new pseudonym of a vehicle is included in the location message given in Equation (11). The location message is queried to the LBS server for the nearest location of interest, as shown in Equation (12).

(9) $$\begin{array}{c} R(\mathrm{PseudoExpiry})=\forall \;i18\in x18\wedge i19\in x19\;|\;(i19[1]\wedge i19[2])\in i18[4]\wedge \\ CheckExpiry(x19^{\prime }:=x19\cup \left\{i19\left[4\right],i19\left[5\right]\right\}). \end{array}$$

(10) $$\begin{array}{c} R(\mathrm{PseudoUpdate})=\forall \;i20\in x20\wedge i21\in x21\;|\;i21[1]\in i20[2]\wedge \\ Update(x21^{\prime }:=x21\cup \left\{i21\left[3\right],i21\left[4\right]\right\}). \end{array}$$

(11) $$\begin{array}{c} R(\mathrm{SetPseudoID})=\forall \;i22\in x22\wedge i23\in x23\;|\;(i23[1]\in i22[2])\wedge \\ Set(x23^{\prime }:=x23\cup \left\{i23\left[3\right]\right\}. \end{array}$$

(12) $$\begin{array}{c} R(\mathrm{SendQuery})=\forall \;i24\in x24\wedge i25\in x25\;|\;(i25[1]\in i24[2]\wedge \\ x25^{\prime }:=x25\cup \left\{i25\left[2\right],i25\left[3\right]\right\}). \end{array}$$

An adversary always tries to obtain the actual location of a target vehicle to know the personal information of a vehicle driver. For this purpose, the adversary captured, the location message communicated with the LBS server, as shown in Equation (13). First, the adversary matches the old pseudonym of a vehicle with the new pseudonym, as shown in Equation (14). Meanwhile, location traces are analyzed in Equation (15).

(13) $$\begin{array}{c} R(\mathrm{CaptureMSG})=\forall \;i26\in x26\wedge i27\in x27\;|\;i27[1]\in i26[2]\wedge \\ Capture(x27^{\prime }:=x27\cup \left\{i27\left[2\right]\right\}. \end{array}$$

(14) $$\begin{array}{c} R(\mathrm{IdMatching})=\forall \;i28\in x28\wedge i29\in x29\wedge i30\in x30\;|\;(i29[1]\wedge i30[1])\\ \;\in i28\left[1\right])\to Compare\left(x30^{\prime }:=x30\cup \left\{i29\left[2\right],i30\left[2\right]\right\}\right). \end{array}$$

(15) $$\begin{array}{c} R(\mathrm{LOCTraces})=\forall \;i31\in x31\wedge i32\in x32\;|\;(i32[1]\in i31[1])\to \\ Match(x32^{\prime }:=x32\cup \left\{i32\left[3\right]\right\}. \end{array}$$

The adversary captures location messages communicated with the LBS server and tries to identify a target vehicle pseudonyms and location tracks. The proposed scheme consists of semantic grouping, location obfuscation, and a pseudonym-changing process, which increases confusion for an adversary that wants to extract the location tracks of vehicles on the road network. Henceforth, HLPN verifies the validity of the proposed scheme and shows the correctness of data flow during the processing of the scheme.

## 6. Experimental Evaluation

This section contains details about the simulation setup, evaluation criteria, and performance comparison of our proposed scheme with existing research work.

### 6.1. Simulation Parameters and Evaluation Criteria

**Figure 6:**
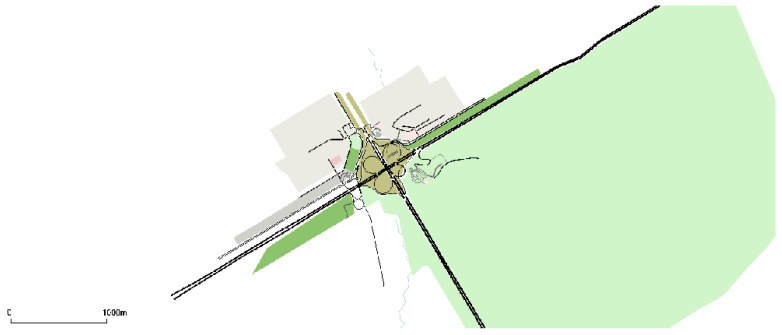
SUMO and OpenStreet Map real-world scenario.

Network Simulator 2 (NS2) is used for the simulation of the proposed scheme. Simulation parameters are given in Table 4. We used SUMO and OpenStreetMap for conducting real-world scenarios of 200 vehicles on the road network. SUMO generates a realistic mobility of vehicles, and OpenStreetMap produces real-world scenarios, as shown in Figure 6. The map is converted into SUMO on a real-world map for vehicle traffic generation. The simulation is run for 400 s.

For the evaluation of location privacy, we used the anonymity set size, entropy, and location traceability as evaluation criteria. ASS is the set of users with similar statuses in which a target user is indistinguishable from the group of users. ASS measures how much the identity of vehicles in a group is protected from an adversary. Its values affect the privacy of vehicles in the network. The higher the ASS, the higher the protection level of privacy will be. The entropy measures the degree of uncertainty in the location information to create uncertainty for an adversary to link pseudonyms of vehicles at different visited locations. Location traceability is the probability used by an adversary in finding the actual routes of a target vehicle in a vicinity. Location traceability is inversely proportional to the location privacy protection level. A lower value of location traceability means a higher protection level of location privacy of vehicles in a region of interest for an adversary.

### 6.2. Performance Comparison

For the performance comparison, most research works take Anonymity Set Size (ASS), entropy and location traceability as evaluation criteria, as discussed in Section 6.1. The simulation results of the proposed scheme SGO are compared with existing Multi-Level Obfuscation Scheme (MLPS) [17] and Virtual Location (VL) scheme [18] in terms of vehicle anonymization, mean entropy, and location traceability. MLPS [17] selects four random position coordinates in connection with neighbors and sends four location messages to the LBS server, which increases computation and communication costs. Moreover, a lot of dummy location messages are generated that impact road network applications. VL [18] takes a neighboring vehicle as a virtual shadow to send multiple requests to the LBS server to hide the actual location of a target vehicle. However, these requests contain the same pseudo-identity and timestamp that provide a way for an adversary to identify a target vehicle.

**Figure 7:**
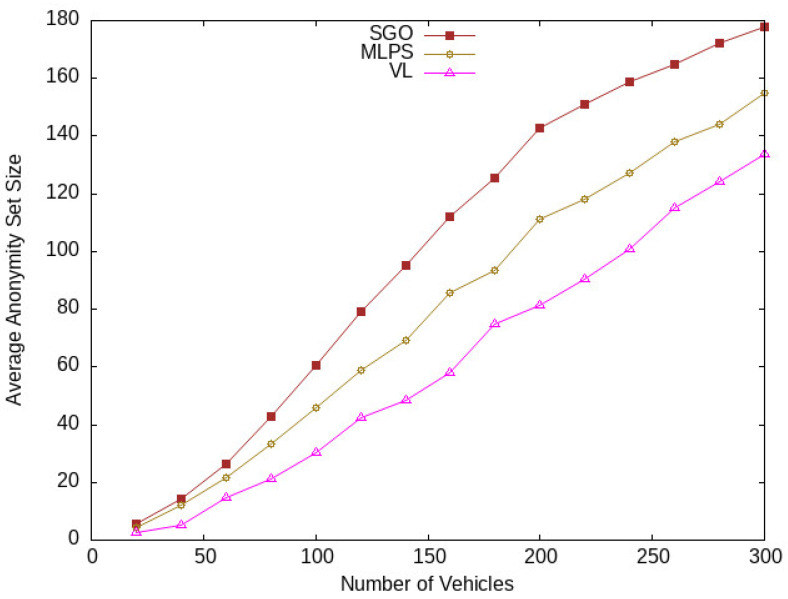
Anonymization of vehicles at different traffic densities.

**Figure 8:**
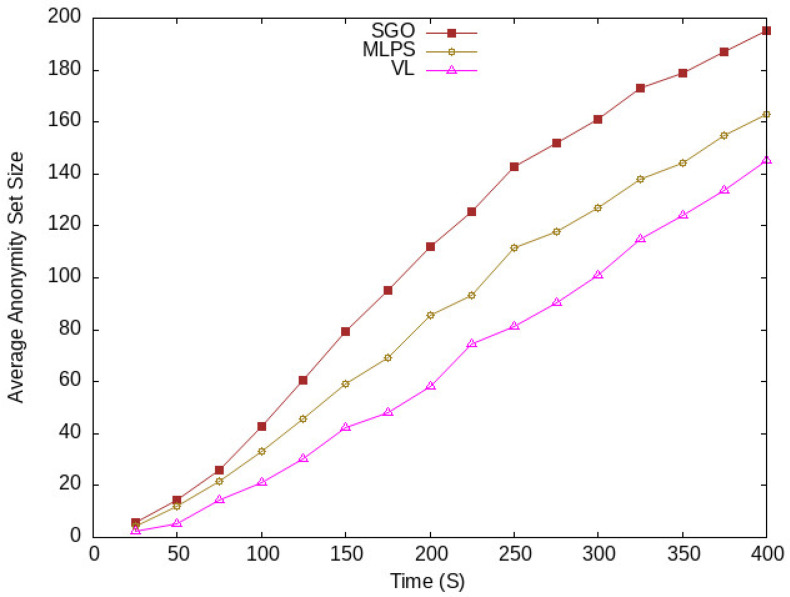
Average anonymity at different time periods.

The average ASS with respect to vehicle traffic density is shown in Figure 7. The proposed scheme SGO shows higher anonymity than existing schemes, which increases confusion for an adversary that wants to identify a target vehicle on the road. Similarly, in Figure 8, the anonymity of vehicles taken in different periods of time also has improved the anonymity of vehicles compared with other schemes. The reason for this improvement in vehicle anonymization is due to the efficient management of vehicles in the semantic groups to obfuscate their location and identity in a vicinity on a road. The LBS server receives location messages in a group manner; each message contains the same position that hides the actual location of vehicles. Meanwhile, the pseudonym-changing approach reduces the linking of the old pseudonym of a vehicle with a new one.

**Figure 9:**
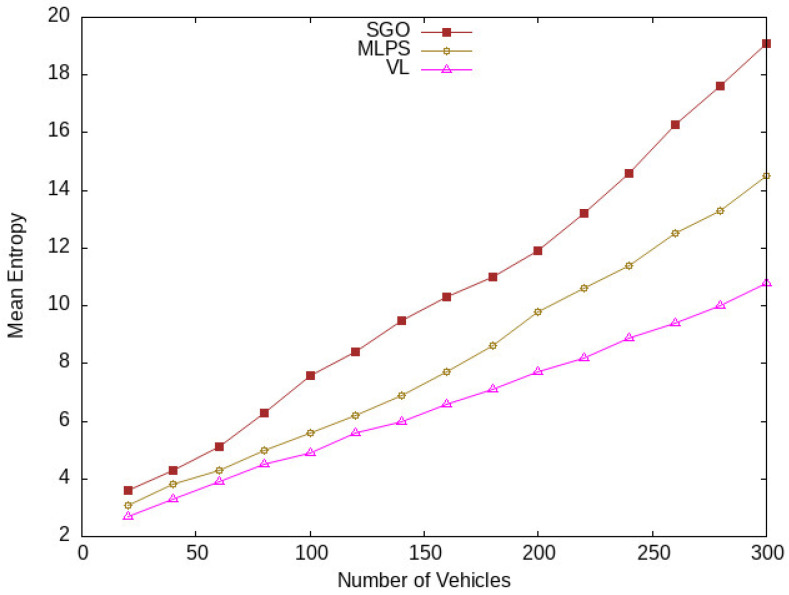
Entropy of vehicles at different traffic conditions.

**Figure 10:**
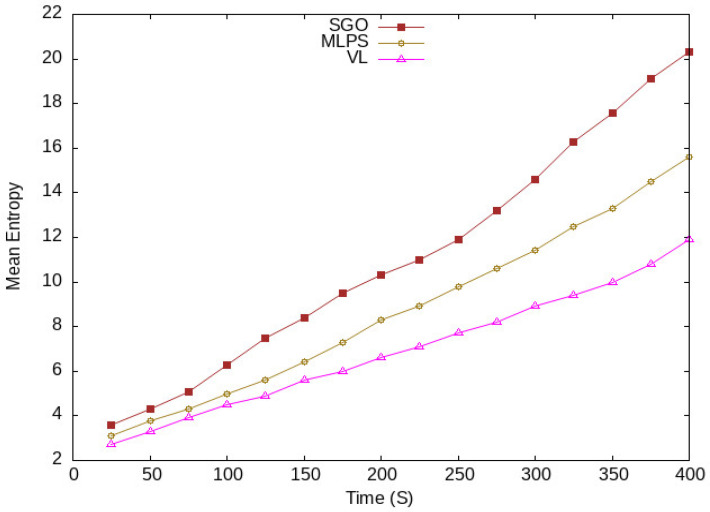
Vehicles mean entropy at different periods.

Entropy shows the degree of randomness of vehicles. That increases uncertainty for an adversary to find out the identity of a target vehicle. The higher the value of entropy, the higher the level of location privacy. The mean entropy results are shown in Figure 9 and Figure 10 with respect to vehicle density and time spent in the network. The mean entropy is considered as the average entropy. As shown in Figure 9, at the start, there is a lower value of mean entropy, but after some time, its value increases with an increase in the vehicle’s density. Similarly, in Figure 10, the value of entropy is increasing with the passage of time in the network. Our proposed scheme SGO shows improvement in the entropy of vehicles compared with [17,18]. There are two reasons behind this improvement: the first one is the higher anonymization of vehicles in the network, and the second one is the usage of semantic location for each member of a group, which hides the actual location of member vehicles in that region. The MLPS [17] only takes random locations not using a pseudonym-changing process, which reduces uncertainty. The VL scheme [18] selects a maximum of two vehicles as a virtual shadow but does not utilize a pseudonym-changing process and uses the same pseudo-identity in all three location messages, which provides a chance for an adversary to extract the identity and location information of a target vehicle. Meanwhile, our proposed scheme SGO considers both location obfuscation and a pseudonym-changing process that increases uncertainty for an adversary to identify a target in the semantic group.

**Figure 11:**
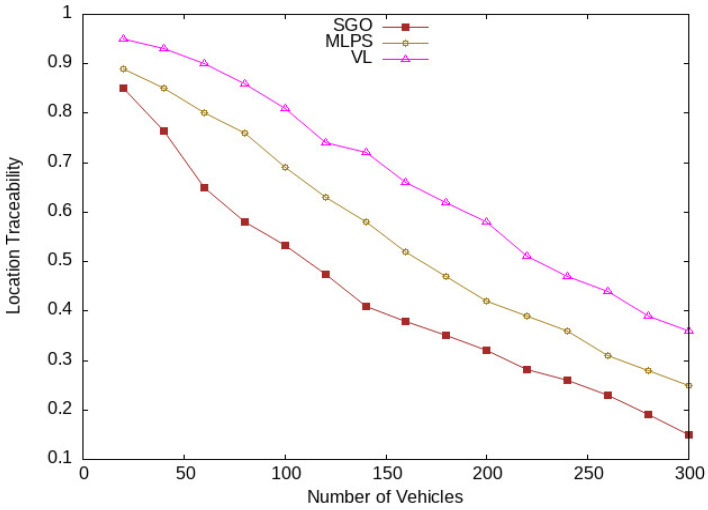
Vehicles tracking at different traffic densities.

**Figure 12:**
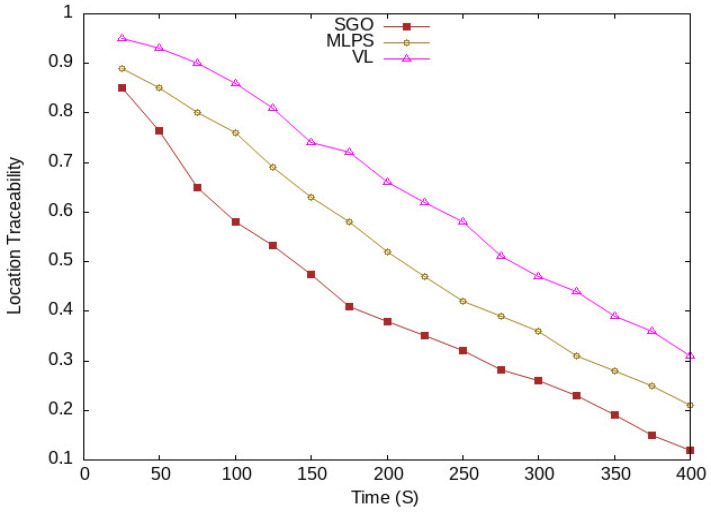
Location tracking at different periods.

The location traceability of vehicles concerning vehicle traffic densities is shown in Figure 11. It is clear from the figure that the proposed scheme lowers the location traceability of vehicles compared with the existing schemes, MLPS [17] and VL [18]. The reason for this improvement is higher vehicle anonymization in the vicinity. A higher number of vehicles are taking part in the pseudonym-changing process, which hides the vehicle’s identity and location. In Figure 12, vehicle location tracking at different periods of time is shown. With the start of the network, the rate of location traceability of vehicles is high; over time, it reduces with the increasing number of vehicles in the network. The proposed scheme SGO still has lower location traceability results compared with MPLS and VL. Again, the improvements in the location traceability results of SGO are organizing vehicles in semantic groups and an obfuscation mechanism that improves the anonymity of vehicles in a semantic group, which reduces the tracking ratio of vehicles. We used both location obfuscation and pseudonym-changing processes. The location obfuscation obscures the location of vehicles, while the pseudonym-changing process covers the actual identity of vehicles. These mechanisms hide the sensitive information of each group member that increases uncertainty for an adversary to identify a vehicle in the region of interest.

## 7. Analysis and Discussion

The proposed scheme is analyzed on the basis of protection against the adversary, impact on location service quality, algorithm complexity, and computation cost, which are discussed in the following.

### 7.1. Protection against Adversary

**Figure 13:**
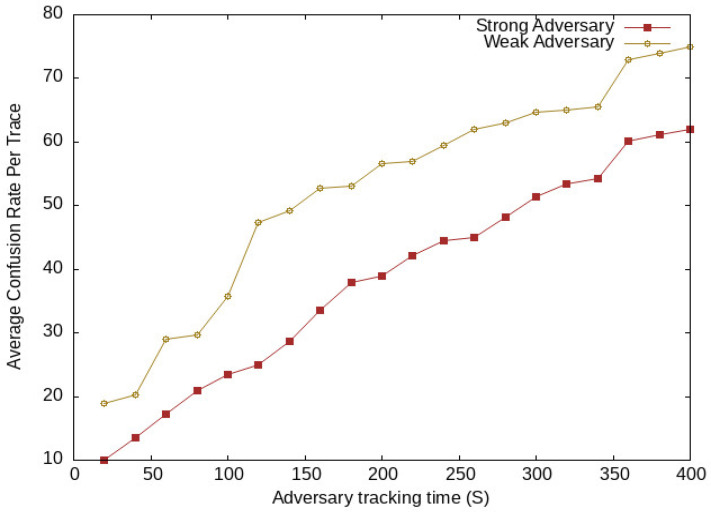
Adversary confusion at different periods.

**Figure 14:**
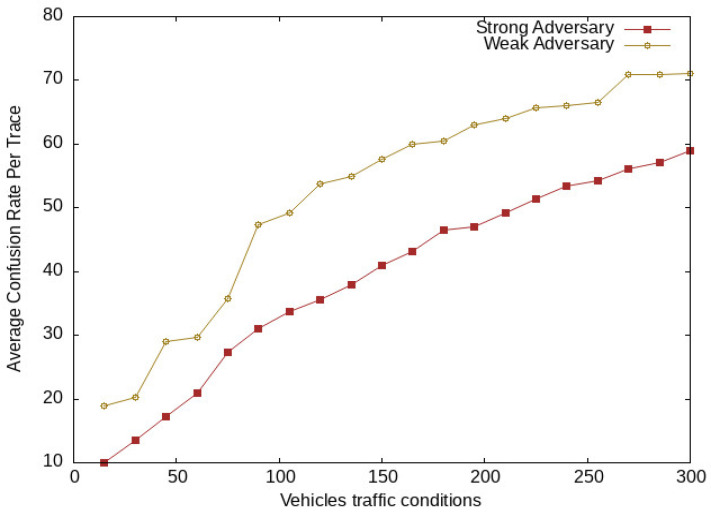
Adversary confusion at different vehicles traffic conditions.

We take a global passive adversary that uses a low-cost transceiver for capturing location messages communicated with the LBS server. The location message contains vehicle pseudo-identity and location information. The adversary analyzes these messages during communication and tries to match the pseudo-identities of a vehicle at different location spots. Here, we take two categories of adversaries, i.e., weak adversaries and strong adversaries. The strong adversary has additional past information about a vehicle. The additional information may be frequently visited locations, used pseudonyms, and locations of interest. This information increases the strength of the adversary for the identification of vehicles located in a vicinity, while the weak adversary has no additional knowledge or information about vehicle location data. We analyze the confusion rate generated by the proposed scheme against the adversary. Here, we used Average Confusion Rate (ACR), which means the average of confusion generated for an adversary by SGO at various periods and vehicle traffic conditions. Figure 13 shows the average confusion generated at different tracing times of both adversaries, while the adversary confusion per trace for various vehicle traffic conditions is shown in Figure 14. At the start of the network, there is a lower confusion rate for both adversaries; after some time, it increases with an increase of vehicle density. There was higher confusion for weak adversaries compared with that for strong adversaries. The proposed scheme SGO still generates uncertainty for both adversaries, which is due to the efficient management of vehicles in semantic groups to obfuscate their locations. This creates confusion for an adversary to extract the actual location of vehicles.

### 7.2. Privacy Impact on Location Service Quality

Protecting the privacy of vehicles moving on the road has some impact on service quality. The concern of privacy schemes is to increase uncertainty for an adversary by hiding the actual location or identity of a vehicle. Meanwhile, the quality of services means providing more and more facilitation services to a vehicle driver. For location privacy, confusion or dummy data are added to location messages to divert the attention of an adversary; however, it impacts the location service quality. The proposed scheme SGO provides efficient location privacy protection and also keeps a lower loss of location service quality compared with existing schemes. For location protection, we use semantic location coordinates which are taken from the actual position coordinates of the road that did not impact location service quality. We take location confusion in terms of position coordinates [3]. The expectation of location perturbation can be calculated as follows.

(16) $$\begin{array}{c} E\left(LOC\right)=\frac{1}{MN}\sum_{i=1}^{M}\sum_{x=1}^{N}P_{i}^{\left(x\right)}\left[\left(LOC_{i}^{\left(x\right)},PID_{i}^{\left(x\right)}\right)V_{i}\right]. \end{array}$$

where LOC is the location of vehicles $V_{i}$ exchanged with the LBS server for the nearest location of interest. *M* is the number of vehicles taking part in the location obfuscation process, *N* is the observation time of a vehicle’s location and $P_{i}$ is an adversary probability to extract the location of vehicles. $PID_{i}$ is the pseudo-identity of vehicles *i*. The quality of services (QoS) depends on accurate location coordinates; the higher the confusion in locations, the lower the quality of location services will be [3]. The QoS is defined in terms of location error as given below.

(17) $$\begin{array}{c} E\left(QoS\right)=\frac{1}{MN}\sum_{i=1}^{M}\sum_{x=1}^{N}[D_{\left(R\right)}\left\{\left(LOC_{i}^{\left(x\right)},PID_{i}^{\left(x\right)}\right),\left(LOC_{i}^{\prime (x)},PID_{i}^{\prime (x)}\right)\right\}]V_{i}. \end{array}$$

where $D_{\left(R\right)}$ distance ranges are taken for the position coordinates of vehicles $V_{i}$, $LOC_{i}^{\prime }$ represents the semantic location coordinates and $PID_{i}^{\prime }$ is the updated pseudonym to be shared with the location server. In our proposed scheme, the dummy location is taken from the actual road environment in lower distance ranges, which reduces the impact on location service quality.

### 7.3. Algorithm Complexity

We evaluated the complexity of the algorithms of the proposed scheme, which is discussed in the following.

#### 7.3.1. Semantic Obfuscation Algorithm

The target vehicle initializes the obfuscation algorithm by finding random position coordinates in different ranges. Let $POS_{n}$ be the cost of finding coordinates in distance ranges and $V_{T}$ be the target vehicle; then, the computation cost of finding position coordinates is $O(POS_{n},V_{T})$. Let $LM_{n}$ be the location messages generated by neighboring vehicles $NV_{n}$; then, the communication complexity of sending these messages to the LBS server is $O(LM_{n},NV_{n})$.

(18) $$\begin{array}{c} Time\;complexity\left(SemanticObfuscation\right)=O\left(POS_{n},V_{T}\right)+O\left(LM_{n},NV_{n}\right)\\ =O\left(POS_{n}+LM_{n}\right)V_{n}\\ =O(n) \end{array}$$

#### 7.3.2. Semantic Grouping

We take the neighbor selection and joining of neighbors processes for the semantic grouping algorithm complexity. Let $V_{n}$ be the number of vehicles taking part in the making of groups with minimum distance range $D_{n}$. The complexity of the neighbor selection process is $O(D_{n},V_{n})$. The joining of vehicles in the groups requires verification. Let $VF_{n}$ be the cost of the vehicle verification process. Then, the vehicle joining process complexity is $O(VF_{n},V_{n})$. The time complexity of the semantic grouping algorithm is:

(19) $$\begin{array}{c} Time\;complexity\left(SemanticGrouping\right)=O\left(D_{n},V_{n}\right)+O\left(VF_{n},V_{n}\right)\\ =O\left(D_{n}+VF_{n}\right)V_{n}\\ =O(n) \end{array}$$

#### 7.3.3. Pseudonym-Changing Process

For the complexity of the pseudonym-changing process, we take the vehicle message broadcast process and pseudonym update process. Let $MSG_{n}$ be the message broadcast in the vicinity by vehicles $V_{n}$ for the pseudonym-changing process; then, the time complexity of this process is $O(MSG_{n},V_{n})$. Let $PID_{n}$ be the pseudonym and $V_{n}$ be the number of vehicles taking part in the pseudonym update process. Then, its complexity is $O(PID_{n},V_{n})$. The total time complexity of pseudonym-changing protocol is given below:

(20) $$\begin{array}{c} Time\;complexity\left(PseudoUpdate\right)=O\left(MSG_{n},V_{n}\right)+O\left(PID_{n},V_{n}\right)\\ =O\left(MSG_{n}+PID_{n}\right)V_{n}\\ =O(n) \end{array}$$

### 7.4. Computation and Communication Cost

**Figure 15:**
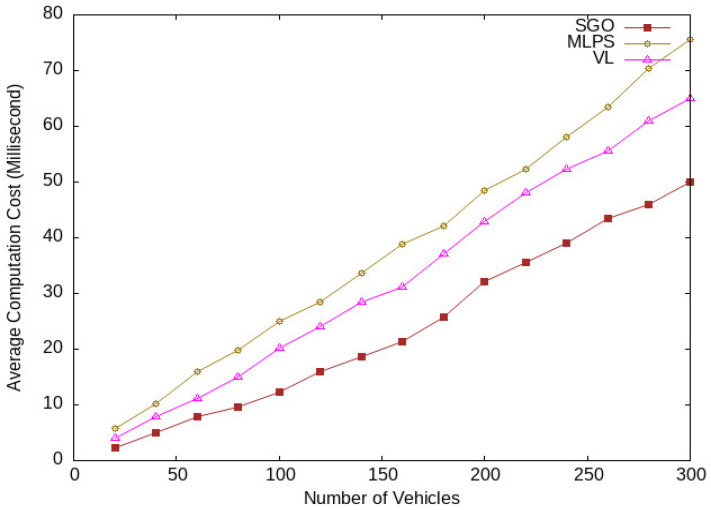
Computation latency for different numbers of vehicles.

Our main concern in this research work is to improve the privacy protection level; however, while designing a privacy-preserving scheme, we should take into account the cost of computation and communication. The computation cost includes location message generation in the group to be sent to a location-based server. The cost of computation of our proposed scheme SGO is lower than the MLPS [17] and VL [18], as shown in Figure 15. MLPS takes four random position coordinates to be included in the location message, which creates extra overheads in the network, and this overhead increases with increases in the vehicle traffic density. That is why MPLS has a higher computation cost compared with SGO and VL. VL selects two neighboring vehicles as a virtual shadow and prepares three location messages to be communicated with the LBS server. This increases its computation cost. The SGO considers a single location message for each group member, which lowers its computation cost.

**Figure 16:**
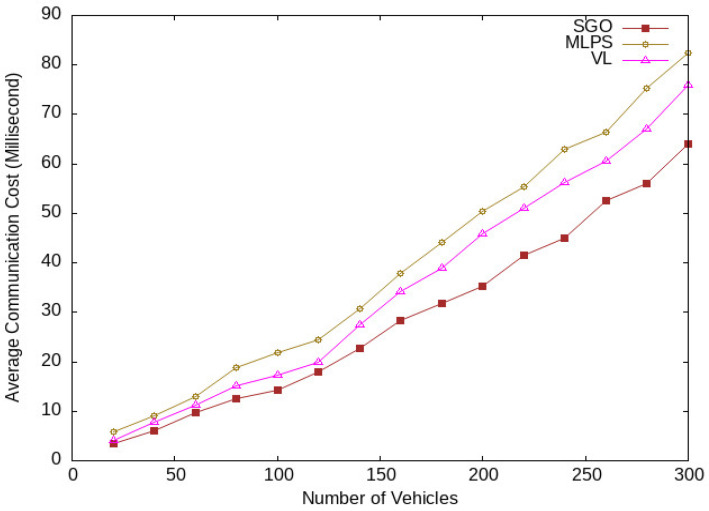
Communication latency for various vehicle traffic conditions.

The communication cost consists of the time required to communicate location messages with group members. The average communication cost of the proposed scheme is lower than MPLS and VL, as given in Figure 16. Again, MLPS takes four random position coordinates, and a single vehicle transmits four location messages to the LBS server, which increases the communication latency. Meanwhile, VL prepares three location messages for communication with a location server that increases communication costs. The proposed scheme SGO uses a single location message, which reduces the communication cost.

### 7.5. Discussion

A vehicle is required to share its location with the LBS server in order to obtain the nearest location of interest. The shared location may be captured by an adversary and create danger for a vehicle driver. There is a need for an efficient mechanism that provides vehicle location protection as well as identity protection. Based on the simulation results, the proposed scheme SGO improves location privacy compared with existing schemes [17,18]. These achievements are due to the management of vehicles in semantic groups, the semantic location concept, and an efficient pseudonym-changing process, which improves vehicle anonymization and entropy as well as reduces location traceability. If we look at the cost of computation and communication results, the proposed scheme SGO reduces it compared with existing schemes [17,18]. The existing schemes select multiple neighboring vehicles as virtual shadows and communicate several dummy location messages with the LBS server, which increases computation and communication costs. The location service quality is not compromised in our case; we used actual location coordinates of the road network in the semantic location messages, which reduces the impact on quality of service. However, the existing schemes use redundant dummy location data, which impacts the quality of service utility. Consequently, the proposed scheme SGO improves location privacy, reduces computation and communication costs, and lowers the impacts on the quality of services in vehicular communication in comparison with existing schemes [17,18].

## 8. Conclusions

We proposed a new semantic location obfuscation technique for preserving the location information of vehicles communicating with the LBS server for the nearest location of interest. In this scheme, vehicles make a semantic group based on the transmission range of neighboring vehicles. Random position coordinates are taken from three different distance ranges. One of the position coordinates is selected as a semantic location which is included in the location message of each group member. The group members communicate with the LBS server using the same location in messages, which protects the actual location information of vehicles. We also used a pseudonym-changing process to update vehicle pseudonyms that hide the actual identity of vehicles. The simulation results show that the proposed scheme SGO achieves improvements in vehicle anonymization and entropy, and it also reduces location traceability and overheads in the network compared with existing schemes. In the future, we are planning to consider a single pseudonym in the location message for each group member, which further increases the anonymity of vehicles in a region of interest. 

## Figures and Tables

**Table 1:** Symbols and their meaning.

Symbols	Meaning
$V_{i}$	Any vehicle i moving on a road
$SP_{R}$	Vehicle speed range
$V_{ID}$	Vehicle identification number
*D*	Vehicle moving direction
CountID	Counting the number of vehicles in a region
*R*	Distance range in meters
LoI	Location of interest
RandPOS	Random position coordinates
SemPOS	Semantic position coordinates
PR	Position coordinates in ranges
$T_{x}$	Transmission range of vehicles
NieghThreshold	Neighbor threshold
PseudoExpiry	Vehicles pseudonym expiry
PseudoIDs	Pseudonyms of vehicles
*T*	Timestamp
POS	Current position of a vehicle

**Table 2:** Symbols used in HLPN for SGO scheme.

Symbol	Description
DistCal	Calculation of distance between neighboring vehicles
NT	Neighbor threshold
NCount	Neighbor count
ThreshSatisfy	Satisfying of neighbor threshold
ThreshFail	Failure of neighbor threshold
SemGroup	Semantic grouping of vehicles
DistRanges	Distance ranges in meters
SemPOS	Semantic position
PC	Pseudonym changing
UpdatedID	Update pseudonyms of vehicles
LocMSG	Location message
SetPOS	Setting semantic position in location message
LOCTraces	Location traces of vehicles

**Table 3:** Places used in HLPN for SGO scheme.

Symbol	Description
φ (Reg-Request)	$P(V_{ID}\times LPN)$
φ (TA)	$P(V_{ID}\times LPN\times PU_{i,k}\times PR_{i,k})$
φ(DistCal)	$P(P_{ID}\times Dist\left(V_{i}\times V_{j}\right))$
φ(NT)	$P(P_{ID}\times MinDist\left(V_{i}\times V_{j}\right)\times NCount)$
φ(Grouping)	$P(P_{ID}\times V\left(i\right)\times NeighThreshold\times AddMembers)$
φ(Reduction)	$P(P_{ID}\times V\left(i\right)\times NeighThreshold\times ReduceMembers\times AddMembers)$
φ(SemGroup)	$P(P_{ID}\times V\left(i\right)\times AddMembers\times Group\left(i\right))$
φ(DistRanges)	$P(P_{ID}\times V\left(i\right)\times R_{1}\times R_{2}\times R_{3}\times Group\left(i\right))$
φ(SemPOS)	$P(P_{ID}\times V\left(i\right)\times Group\left(i\right)\times Random\left(Position\right))$
φ(PC)	P(V(i)×Group(i)×PseudoID(i)×Expiry×flag(i))
φ(UpdatedID)	P(V(i)×Group(i)×PseudoID(i)×flag(i))
φ(LocMSG)	P(V(i)×Group(i)×PseudoID(i)×Random(Position))
φ(LBS)	P(V(i)×PseudoID(i)×Random(Position))
φ(Anaylsis)	P(V(i)×LocMsgs(PseudoID,SemPosition))
φ(OldPseudo)	P(V(i)×OldPseudoID(i)×LOC)
φ(Identification)	P(V(i)×PseudoID(i),SemPosition)

**Table 4:** Simulation parameters.

Parameters	Values
Simulator	NS-2, SUMO
Map	OpenStreetMap
Area	5623 × 5267 m
Number of vehicles	300
Vehicle speed	0–15 m/s
Transmission range	500 m
Routing protocol	AODV
Mobility model	Random Waypoint
Simulation time	400 s

## Data Availability

Data are contained within the article.
